# Calpain Activator Dibucaine Induces Platelet Apoptosis

**DOI:** 10.3390/ijms12042125

**Published:** 2011-03-25

**Authors:** Weilin Zhang, Jun Liu, Ruichen Sun, Lili Zhao, Juan Du, Changgeng Ruan, Kesheng Dai

**Affiliations:** 1 School of Biological Science and Medical Engineering, Beijing University of Aeronautics and Astronautics, 37 Xueyuan Road, Haidian District, Beijing 100083, China; E-Mails: zhangwl@be.buaa.edu.cn (W.Z.); liujuno0o@sina.com (J.L.); ruichen-sun@hotmail.com (R.S.); loveenly1314@hotmail.com (L.Z.); djsmile1987@yahoo.com.cn (J.D.); 2 The First Affiliated Hospital of Soochow University, Jiangsu Institute of Hematology, Key Laboratory of Thrombosis and Hemostasis, Ministry of Health, Suzhou, 215007, China; E-Mail: changgengruan@hotmail.com

**Keywords:** platelets, calpain, dibucaine, platelet apoptosis, platelet activation

## Abstract

Calcium-dependent calpains are a family of cysteine proteases that have been demonstrated to play key roles in both platelet glycoprotein Ibα shedding and platelet activation and altered calpain activity is associated with thrombotic thrombocytopenic purpura. Calpain activators induce apoptosis in several types of nucleated cells. However, it is not clear whether calpain activators induce platelet apoptosis. Here we show that the calpain activator dibucaine induced several platelet apoptotic events including depolarization of the mitochondrial inner transmembrane potential, up-regulation of Bax and Bak, down-regulation of Bcl-2 and Bcl-X_L_, caspase-3 activation and phosphatidylserine exposure. Platelet apoptosis elicited by dibucaine was not affected by the broad spectrum metalloproteinase inhibitor GM6001. Furthermore, dibucaine did not induce platelet activation as detected by P-selectin expression and PAC-1 binding. However, platelet aggregation induced by ristocetin or α-thrombin, platelet adhesion and spreading on von Willebrand factor were significantly inhibited in platelets treated with dibucaine. Taken together, these data indicate that dibucaine induces platelet apoptosis and platelet dysfunction.

## 1. Introduction

Calpains are a family of calcium-dependent cysteine proteases that play key roles in both primary cascades of platelet activation such as platelet aggregation, adhesion, spreading, and late events of platelet-dependent fibrin clot retraction and thrombus formation [1–4]. We reported recently that calpains play critical roles in a disintegrin and metalloproteinase 17 (ADAM17) mediated glycoprotein (GP) Ibα shedding and the negative regulation of the surface expression of the functional receptor [5]. In particular, altered calpain activity is associated with thrombotic thrombocytopenic purpura (TTP), an unusual but potentially life-threatening disease, whereas the pathogenesis of thrombocytopenia is poorly understood [6,7]. Calpain activators induce apoptosis in several types of nucleated cells [8–10]. Up to now, calpains have been reported to be associated with GPIbα shedding and platelet activation, however, it is still unclear whether calpain activator dibucaine induces platelet apoptosis.

Platelet apoptosis elicited by physiological or chemical compounds, pathological high shear stress, or platelet storage occurs widely *in vitro* and *in vivo* [11–15]. Platelet apoptosis generally arises from the intrinsic mitochondrial pathway characterized by depolarization of mitochondrial inner transmembrane potential (ΔΨm), increased expression of pro-apoptotic proteins Bak and Bax, decreased expression of anti-apoptotic proteins Bcl-2 and Bcl-X_L_, caspase-3 activation, and phosphatidylserine (PS) exposure [11–15]. Although some of the platelet apoptosis events are similar to those of platelet activation with respect to morphological and biochemical characteristics, the signaling pathways leading to platelet apoptosis and platelet activation are distinct from each other, indicating that platelet activation and apoptosis occur independently under physiological conditions [16–21]. During platelet apoptosis induced by calcium ionophore A23187 or the physiological agonist α-thrombin, both calpains and caspase-3 are activated and cleave substrates such as the cytoskeletal regulatory proteins gelsolin and fodrin [11,22–24]. However, the interaction between these two proteases in platelet apoptosis remains controversial.

In the present study, the data demonstrate that the calpain activator dibucaine induces human platelet apoptosis independent of GPIbα shedding. Dibucaine does not induce platelet activation, however, it obviously inhibits platelet function.

## 2. Materials and Methods

### 2.1. Antibodies and Reagents

Monoclonal antibodies SZ2 against GPIbα and SZ51 against P-selectin were generous gifts from Dr. Changgeng Ruan (Soochow University, Suzhou, China). Purified human VWF and botrocetin were generous gifts from Dr. Xiaoping Du (University of Illinois, Chicago, IL, USA). Ristocetin, α-thrombin, aprotinin, dimethyl sulfoxide (DMSO), anti-human gelsolin antibody, FITC-conjugated PAC-1, and dibucaine were purchased from Sigma (St. Louis, MI, USA). Non-essential amino acids, penicillin and streptomycin, L-glutamine, and L-trans-Epoxysuccinyl-leucylamido (4-guanidino) (E64) were purchased from Roche Molecular Biochemicals (Indianapolis, IN, USA). Caspase-3 inhibitor z-DEVD-fmk was purchased from Bender Medsystem (Vienna, Austria). Calpain inhibitor carbobenzoxy-valinyl-phenylalaninal (MDL28170), calpain inhibitors I and II, A23187 and GM6001 were purchased from Calbiochem (San Diego, CA, USA). Goat anti-mouse immunoglobulin (IgG) conjugated with horseradish peroxidase (GAM-HRP), goat anti-rabbit immunoglobulin (IgG) conjugated with horseradish peroxidase (GAR-HRP), FITC-conjugated goat anti-mouse IgG (FITC-GAM), and Alexa Fluor 488-conjugated goat anti-mouse IgG were purchased from Biosource (Camarillo, CA, USA). Anti-actin antibody was from Boehringer Mannheim Biochemicals (Indianapolis, IN, USA). The anti-tubulin antibody was purchased from NeoMarkers (Fremont, CA, USA). The mitochondria isolation kit was purchased from Pierce (Rockford, IL, USA). The anti-COX-1 antibody was purchased from MitoSciences (Eugene, OR, USA). Monoclonal antibodies against Bax, Bak, Bcl-2, Bcl-X_L_ and caspase-3 were purchased from Santa Cruz Biotechnology (Santa Cruz, CA, USA).

### 2.2. Platelet Preparation

For studies involving human subjects, approval was obtained from the Beihang University institutional review board. Informed consent was provided according to the Declaration of Helsinki. Blood collection and preparation of washed platelets were performed as previously described [17].

### 2.3. Platelet Aggregation Assay

For platelet aggregation studies, washed platelets were incubated with dibucaine (500 μmol/L), and then platelet aggregation was induced by the addition of ristocetin (1.25 mg/mL) plus VWF (35 μg/mL) or α-thrombin (0.1 U/mL) and measured by a turbidometric platelet aggregometer (Xinpusen, Beijing, China) at 37 °C with a stirring speed of 1000 rpm [18].

### 2.4. ΔΨm Measurement

Washed platelets (3 × 10^8^ /mL) were incubated with dibucaine (125 μmol/L, 250 μmol/L, 500 μmol/L) at RT for 15 min, ΔΨm was determined using the potential sensitive dye tetramethyl-rhodamine-ethylester (TMRE). Briefly, TMRE was added into the dibucaine pre-treated platelets to a 100 nmol/L final concentration. Then samples were further incubated in the dark at 37 °C for 20 minutes (min) and analyzed by flow cytometry. TMRE signals were excited using a 488 nm krypton-argon laser line and emissions were captured using filters at 625 nm. In some experiments, platelets were pre-incubated with GM6001 (100 μmol/L) at RT for 10 min, and then incubated with dibucaine (500 μmol/L) at RT for 15 min and subjected into ΔΨm measurement [18].

### 2.5. PS Externalization Assay

Washed platelets (3 × 10^8^/mL) were incubated with dibucaine (125 μmol/L, 250 μmol/L, 500 μmol/L) at RT for 15 min. Annexin V binding buffer was mixed with pre-treated platelets and annexin V-FITC at a 50:10:1 ratio. Samples were gently mixed and incubated at RT for 15 min in the dark, then analyzed by flow cytometry. In some experiments, platelets were pre-incubated with GM6001 (100 μmol/L) at RT for 10 min, and then incubated with dibucaine (500 μmol/L) at RT for 15 min and subjected into PS exposure analysis [18].

### 2.6. Platelet Surface Staining

Washed platelets (3 × 10^8^/mL) were incubated with dibucaine (500 μmol/L) at RT for 15 min. To analyze P-selectin surface expression, platelets pre-treated with dibucaine were firstly incubated with SZ51 at RT for 30 min, and then further incubated with FITC-GAM in the dark at RT for 30 min. The treated platelets were analyzed by flow cytometry. For PAC-1 binding, platelets pre-treated with dibucaine were incubated with FITC-labeled soluble PAC-1 at RT for 20 min in the dark. Then platelets were fixed with 1% cold paraformaldehyde, further incubated at 4 °C in the dark for 30 min, and analyzed by flow cytometry. A23187 was set as a positive control. As negative controls, platelets were incubated with mouse IgG and then incubated with FITC-GAM [18].

### 2.7. Western Blot Analysis

Washed platelets (3 × 10^8^/mL) were treated with dibucaine (125 μmol/L, 250 μmol/L, 500 μmol/L) at RT for 15 min, and lysed with an equal volume of lysis buffer containing 0.1 mmol/L E64, 1 mmol/L phenylmethylsulfonyl fluoride (PMSF) and 1/100 aprotinin on ice for 30 min, and the whole lysate was resolved by sodium dodecylsulfate polyacrylamide gel electrophoresis (SDS-PAGE) and immunoblotted with anti-gelsolin antibody (1:2500) or anti-caspase-3 antibody (1:1000), respectively. To evaluate Bcl-2 family protein expression, lysate was resolved by SDS-PAGE, and immunoblotted with anti-Bak (1:500), anti-Bax (1:500), anti-Bcl-2 (1:500), or anti-Bcl-X_L_ (1:500), respectively. Anti-actin antibody was used to confirm that protein inputs in each lane were similar. In some experiments, platelets were pre-incubated with GM6001 (100 μmol/L) at RT for 10 min, or calpain inhibitor MDL28170 (100 μmol/L) at RT for 10 min, or caspase-3 inhibitor z-DEVD-fmk (50 μmol/L), or control DMSO at RT for 15 min, and then were further incubated with dibucaine (500 μmol/L) at RT for 15 min and subjected into western blot analysis [18].

In the isolation and analysis of the platelet mitochondrial fraction assay [25], platelets were treated with dibucaine (500 μmol/L) or DMSO at RT for 15 min, and then were suspended in mitochondrion isolation buffer A for 2 min, buffer B for 5 min, and buffer C for 5 min followed by centrifugation. The supernatants were centrifuged at 12,000 g for 20 min. The pellets containing the mitochondria were washed once with buffer C, then were incubated in lysis buffer for 30 min, and centrifuged at 12,000 g for 5 min. The samples were subjected to Western blot analysis.

### 2.8. Confocal Microscopy

Platelets (3 × 10^8^/mL) were pre-incubated with dibucaine (500 μmol/L) or DMSO at RT for 15 min. The Lab-Tek cover slips (Nunc) were pre-coated with 20 μg/mL of VWF and blocked with 5% BSA. Platelets pre-treated with dibucaine were incubated with botrocetin (5 μg/mL) and allowed to adhere to the prepared cover slips as described previously [18]. Platelets were fixed with 4% paraformaldehyde, permeabilized and blocked with BSA. Samples were incubated with SZ2 (10 μg/mL) firstly, and then with Alexa Fluor 488-conjugated goat anti-mouse IgG. Samples were scanned under a confocal microscope (63 × objective lens) (Lab-Tek MicroImaging, Inc. LSM510).

### 2.9. Caspase-3 Activity Assay

Caspase-3 activity was determined using the caspase-3 activity kit (Beyotime Institute of Biotechnology, Haimen, China) as described previously [26]. Briefly, platelets (3 × 10^8^/mL) were pre-treated with caspase-3 inhibitor z-DEVD-fmk (50 μmol/L) or DMSO at RT for 15 min, or calpain inhibitor MDL28170 (100 μmol/L) at RT for 10 min, and then were further incubated with dibucaine (500 μmol/L) at RT for 15 min. Caspase-3 activity assays were performed on 96-well microtitre plates by incubating 10 μL platelet lysate per sample in 80 μL reaction buffer and 10 μL caspase-3 substrate (Ac-DEVD-*p*NA) (2 mmol/L). Samples were incubated at 37 °C for 4 h and were measured with an enzyme-linked immunosorbent assay (ELISA) reader at an absorbance of 405 nm. The specific caspase-3 activity, normalized for total proteins of platelet lysates, was then expressed as fold of the baseline caspase-3 activity of the control DMSO-treated sample.

## 3. Results and Discussion

### 3.1. Dibucaine Dose-Dependently Induces Depolarization of ΔΨm in Platelets

Calcium-mediated activation of calpain induces apoptosis in several types of nucleated cells [9,10]. In particular, it has been reported that ONO-3403 (ethyl *N*-allyl-*N*-[(*E*)-2-methyl-3-[4-(4-amidinophenoxycarbonyl)phenyl]propenoyl]amino acetate methansulfonate), a specific stimulator of calpain, induces apoptosis cascades in NIH3T3 mouse fibroblasts [8], suggesting that the calpain activator dibucaine may induce platelet apoptosis. To test this hypothesis, we first determined the effectiveness of dibucaine, an established calpain activator [27]. Platelets were incubated with dibucaine and subjected to Western blot analysis. The results showed that talin and filamin A, both of which are recognized substrates of calpain, were dose-dependently cleaved in platelets pre-treated with dibucaine (data not shown). Furthermore, cleavage of filamin A was inhibited by calpain inhibitors (Supplementary Figure I), indicating that dibucaine is a calpain-specific activator. In addition, dibucaine was able to induce cleavage of filamin A in the absence of extracellular calcium (Supplementary Figure II), consistent with previous findings [27]. Therefore, dibucaine was selected in the following studies.

**Figure 1.**
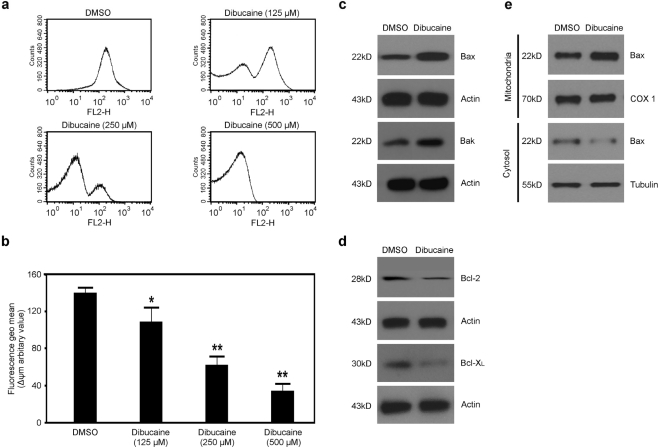
Dibucaine elicits depolarization of ΔΨm, upregulation of Bax and Bak, and downregulation of Bcl-2 and Bcl-X_L_ in platelets. Washed platelets were incubated with dibucaine at RT for 15 min. **(a)** TMRE was added into the platelets pre-treated with dibucaine to a final concentration of 100 nmol/L. Then samples were further incubated in the dark at 37 °C for 20 min and analyzed by flow cytometry; **(b)** Quantitative data from three experiments are shown (mean ± SD). ** *P* < 0.01 compared with DMSO; *P < 0.05 compared with DMSO; **(c,d)** Platelets pre-treated with dibucaine were lysed and subjected to Western blot analysis with anti-Bax, anti-Bak **(c)**; anti-Bcl-2, or anti-Bcl-X_L_ **(d)**; respectively. Actin levels demonstrate equal loading; **(e)** Platelets were incubated with dibucaine at RT for 15 min. Cytosol and mitochondrial fractions were isolated and analyzed by Western blot with anti-Bax as described in Materials and methods. Cytochrome c oxidase subunit 1 (COX 1) (for the mitochondrial fraction) and tubulin (for the cytosolic fraction) were used as respective internal controls. Results are representative of three separate experiments with different donors.

There are two signal transduction pathways leading to apoptosis in nucleated cells: the receptor-mediated apoptotic pathway and the mitochondria-linked apoptotic pathway. However, platelet apoptosis commonly arises from the mitochondria-mediated signaling pathway characterized by depolarization of ΔΨm [17]. So effects of dibucaine on depolarization of ΔΨm in platelets were investigated by flow cytometry with the cell-permeable lipophilic cationic dye TMRE. Figure 1a and b demonstrated clearly that dibucaine, but not the vehicle control DMSO, dose-dependently elicited depolarization of ΔΨm in platelets, as monitored by decreased fluorescence in TMRE-stained platelets, and an increase of depolarized platelets.

### 3.2. Dibucaine Elicits Up-regulation of Bax and Bak, and Down-regulation of Bcl-2 and Bcl-X_L_ in Platelets

Depolarization of ΔΨm occurs in both platelet apoptosis and platelet activation induced by strong agonists [16–21]. Whether depolarization of ΔΨm resulted from platelet apoptosis was further investigated. Although platelets are anucleated cells, the Bcl-2 family proteins, interacting with the mitochondrial outer membrane, are well-characterized regulators of platelet apoptosis and act as a checkpoint upstream of caspases and a mitochondria gateway where a variety of apoptotic signals converge [17,18]. Therefore, to further investigate whether dibucaine can elicit platelet apoptosis through the mitochondria-mediated pathway, the expression profiles of the Bcl-2 family proteins including Bax, Bak, Bcl-2, and Bcl-X_L_ were analyzed. As shown in Figure 1c and d, the expression levels of pro-apoptotic proteins Bax and Bak increased significantly in platelets following dibucaine induction. Conversely, decreased expression levels of anti-apoptotic proteins Bcl-2 and Bcl-X_L_ were detected simultaneously, consistent with previous studies [28]. In addition, there was an obvious increase in mitochondrial membrane-bound Bax.

### 3.3. Caspase-3 Is Activated in Platelets Treated with Dibucaine

**Figure 2.**
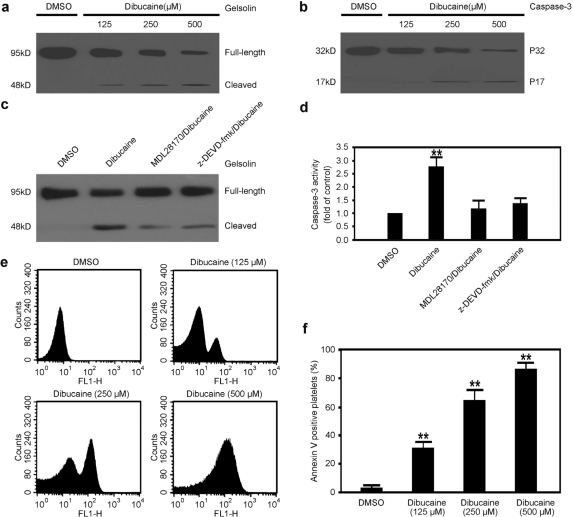
Dibucaine induces gelsolin cleavage, caspase-3 activation and PS exposure in platelets. Washed platelets were incubated with dibucaine at RT for 15 min. **(a,b)** Platelets pre-treated with dibucaine were lysed and subjected to Western blot analysis with anti-gelsolin **(a)**; or anti-caspase-3 **(b)**; respectively. Results are representative of three separate experiments with different donors; **(c,d)** Washed platelets were pre-treated with caspase-3 inhibitor z-DEVD-fmk, or calpain inhibitor MDL28170, or DMSO, and then were further incubated with dibucaine and subjected to Western blot analysis with anti-gelsolin **(c)** or caspase-3 activity analysis **(d)** as described in Materials and methods. Data shown are the mean ± SD (n = 3); **(e)** Annexin V binding buffer was mixed with the dibucaine-pre-treated platelets and annexin V-FITC at a 50:10:1 ratio. Samples were gently mixed and incubated at RT for 15 min in the dark, then analyzed by flow cytometry; **(f)** Mean ± SD of the percentage of PS-positive platelets from three independent experiments is shown. ** *P* < 0.01 compared with DMSO.

It is well known that Bcl-2 family proteins regulate the mitochondrial inner transmembrane potential as well as the release of cytochrome c, which results in the activation of caspases, especially caspase-3, the executioner of apoptosis [17,18]. Furthermore, both calpains and caspase-3 are activated and involved in platelet apoptosis induced by A23187 or α-thrombin [11,22,23]. However, it is still unknown whether calpains function upstream of caspases or not in platelet apoptosis induced by dibucaine. To answer this question, gelsolin, a cytoskeletal regulatory protein and also the confirmed substrate of caspase-3, was employed to analyze the activation state of caspase-3 in Western blot assays. As demonstrated in Figure 2a, dibucaine dose-dependently elicited the presence of the 48 kDa cleaved fragment of gelsolin, indicating that caspase-3 was activated.

Although gelsolin is generally regarded as a specific substrate of the caspase-3, there is a report that calpain also cleaves gelsolin [24]. Therefore, the activation of caspase-3 was further analyzed. As expected, compared with the control samples, the 17 kDa fragment representing caspase-3 activation dose-dependently presented in platelets in response to the treatment of dibucaine (Figure 2b). In addition, activation of caspase-3 was observed in platelets treated with dibucaine, which was significantly inhibited by the caspase-3 inhibitor z-DEVD-fmk or calpain inhibitor MDL28170 (Figure 2d). Although Wolf *et al.* [24] reported that caspase-3 was not activated in platelets treated with thrombin, other groups demonstrated that activation of caspase-3 was induced by thrombin [11,22], which is not in conflict with our findings.

### 3.4. Dibucaine Induces PS Exposure in Platelets

Depolarization of ΔΨm and activation of caspase-3 are the common characteristics of apoptosis in platelets induced by various stimuli [17,18]. In contrast, PS exposure is the characterized morphology of apoptosis in platelets stimulated by strong agonists [19]. Therefore, in order to investigate whether dibucaine is potent to elicit PS exposure during platelet apoptosis, PS exposure was measured in platelets pre-incubated with dibucaine using flow cytometric analysis. A dose-dependent PS exposure was observed in platelets treated with dibucaine (Figure 2c and d), suggesting that dibucaine is a strong apoptosis stimulator. In addition, PS exposure was significantly inhibited by the caspase-3 inhibitor z-DEVD-fmk, further supporting that this process is mediated by caspase-3 (Supplementary Figure III). Together, these data indicate that dibucaine triggers signaling cascades leading to platelet apoptosis.

### 3.5. Dibucaine-Induced Platelet Apoptosis Is Independent of GPIbα Shedding

**Figure 3.**
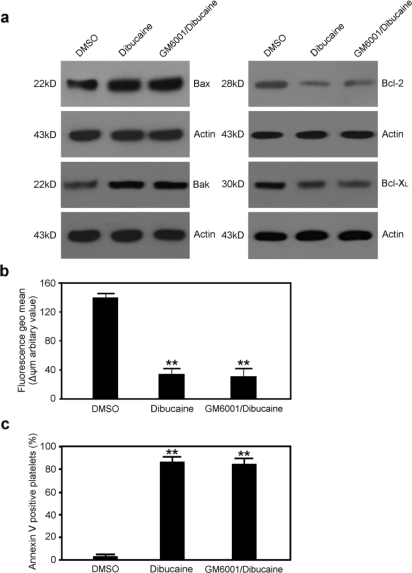
Platelet apoptosis events induced by dibucaine are independent of GPIbα shedding. Platelets were pre-incubated with GM6001, and then further incubated with dibucaine. **(a)** GM6001 and dibucaine treated platelets were lysed and subjected to Western blot analysis with anti-Bax, anti-Bak, anti-Bcl-2, or anti-Bcl-X_L_. Actin levels demonstrate equal loading; **(b)** The mitochondrial inner transmembrane potential in GM6001 and dibucaine treated platelets was analyzed and presented as mean ± SD (n = 3); **(c)** Platelets treated with GM6001 and dibucaine were subjected to phosphatidylserine (PS) exposure analysis by flow cytometry. Quantitation data from three separate experiments are shown as mean ± SD (n = 3). **P < 0.01 compared with DMSO.

We recently reported that calpains play regulatory roles in ADAM17-mediated GPIbα shedding [5]. To determine whether dibucaine-induced platelet apoptosis results from GPIbα shedding, platelets were pre-incubated with GM6001, a recognized ADAM17 inhibitor [5], then further incubated with dibucaine, and markers of the apoptotic cascades were analyzed. GM6001 effectively blocked GPIbα shedding (data not shown), however, it exerted no obvious effect on dicucaine-induced apoptosis events such as depolarization of ΔΨm, expression levels of Bcl-2 family and PS exposure (Figure 3), indicating that dibucaine-induced platelet apoptosis is independent of GPIbα shedding.

### 3.6. Dibucaine Does Not Induce Platelet Activation

**Figure 4.**
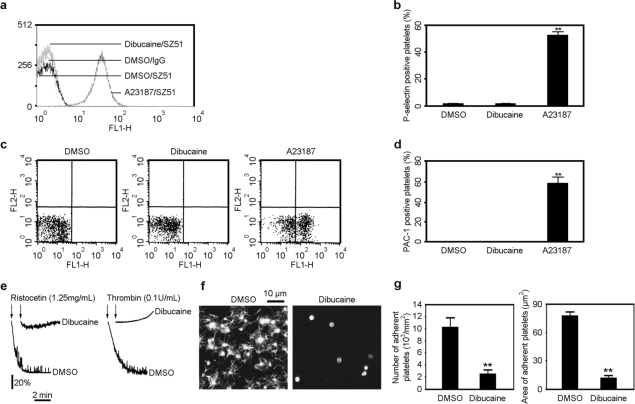
Dibucaine does not induce platelet activation, but it inhibits platelet function. **(a,b,c,d)** Platelets were incubated with dibucaine, DMSO, or A23187. P-selectin surface expression was analyzed by flow cytometry with SZ51 as the antibody. Representative histograms from three independent experiments are shown **(a)** Quantitation data from three separate results are shown as mean ± SD **(b); (c,d)** Platelets pretreated with dibucaine, DMSO, or A23187 were analyzed the PAC-1 binding by flow cytometry with FITC-conjugated PAC-1. Typical dot blots representing PAC-1 binding from three different experiments are present **(c)** Quantitation data from three separate experiments are shown as mean ± SD **(d)** **P < 0.01 compared with DMSO; **(e)** Washed platelets were incubated with dibucaine at RT for 15 min, and then platelet aggregation was induced by the addition of ristocetin plus VWF or α-thrombin and measured by a turbidometric platelet aggregometer. Representative results from three different experiments are shown; **(f,g)** Dibucaine pretreated platelets were incubated with botrocetin on the cover slips pre-coated with VWF. After fixation and permeabilization, the cover slips were incubated with SZ2, and then were stained and scanned under a confocal microscope (63 × lens). Typical images of three different experiments are shown **(f)** Numbers of adherent platelets and mean areas of spread platelets from 12 images were quantified and demonstrated as mean ± SD **(g)** **P < 0.01 compared with DMSO.

Our findings demonstrated that dibucaine induces PS exposure in platelets. However, there is also a possibility that PS exposure results from platelet activation [17]. In order to exclude the interference of platelet activation and further confirm that the mechanisms leading to platelet activation and platelet apoptosis are distinct from each other, the effects of dibucaine on platelet activation events were investigated. Platelets were incubated with dibucaine, using DMSO and A23187 as negative and positive controls, respectively, then the surface expression of P-selectin, an established marker of α-granule release and platelet activation, was analyzed. Flow cytometry results demonstrated that dibucaine does not elicit obvious surface expression of P-selectin (Figure 4a and b). To further study whether dibucaine can induce platelet activation, the activation of integrin α_IIb_β_3_ was examined by flow cytometry with FITC-conjugated PAC-1, a confirmed antibody that specifically reacts with the activated integrin α_IIb_β_3_. Figure 4c and d showed that no obvious PAC-1 binding was observed in platelets pre-treated with dibucaine, in contrast, PAC-1 positive platelets presented in A23187 treated samples, excluding the interference of experimental conditions. Taken together, these data suggest that dibucaine does not induce platelet activation.

### 3.7. Dibucaine Impairs Platelet Function

Platelets are critical players in both hemostasis and thrombosis. It has also been proposed that altered calpain activity is involved in TTP [6,7]. Our findings indicated that dibucaine induces platelet apoptosis but not platelet activation. However, it is still unknown whether dibucaine affects platelet function. To answer this question, platelet aggregation and confocal assays were employed to analyze the roles of dibucaine in platelet aggregation, adhesion and spreading on VWF surface. As shown in Figure 4e, both ristocetin/VWF- and α-thrombin-induced platelet aggregations were markedly diminished in dibucaine-incubated platelets. Furthermore, both the number of adherent platelets and the area of platelet membrane spreading on VWF were obviously decreased in platelets in response to dibucaine treatment (Figure 4f and g). Collectively, these data indicate that dibucaine induces platelet dysfunction.

## 4. Conclusions

In the current study, the data show that the calpain activator dibucaine elicits platelet apoptosis independent of GPIbα shedding. Dibucaine does not induce platelet activation, however, it obviously inhibits platelet function. Thus, in addition to the pivotal and proven roles of calpain in GPIbα shedding and platelet activation, the data contribute to the importance of calpain in platelet apoptosis, which might be one cause of TTP.

## References

[1] Hato T, Pampori N, Shattil SJ (1998). Complementary roles for receptor clustering and conformational change in the adhesive and signaling functions of integrin αIIbβ3. J. Cell Biol.

[2] Azam M, Andrabi SS, Sahr KE, Kamath L, Kuliopulos A, Chishti AH (2001). Disruption of the mouse μ-calpain gene reveals an essential role in platelet function. Mol. Cell Biol.

[3] Croce K, Flaumenhaft R, Rivers M, Furie B, Furie BC, Herman IM, Potter DA (1999). Inhibition of calpain blocks platelet secretion, aggregation, and spreading. J. Biol. Chem.

[4] Fox JEB, Reynolds CC, Phillips DR (1983). Calcium-dependent proteolysis occurs during platelet aggregation. J. Biol. Chem.

[5] Wang Z, Shi Q, Yan R, Liu G, Zhang W, Dai K (2010). The role of calpain in the regulation of ADAM-17-dependent GPIbα ectodomain shedding. Arch. Biochem. Biophys.

[6] Murphy WG, Moore J, Kelton JG (1987). Calcium-dependent cysteine protease activity in the sera of patients with thrombotic thrombocytopenic purpura. Blood.

[7] Kelton JG, Warkentin TE, Hayward CPM, Murphy WG, Moore JC (1992). Calpain activity in patients with thrombotic thromocytopenic purpur is associated with platelet microparticles. Blood.

[8] Hiwasa T (1996). Induction of apoptosis by a calpain stimulator, ONO-3403. Apoptosis.

[9] Gil-Parrado S, Fernandez-Montalvan A, Assfalg-Machleidt I, Popp O, Bestvater F, Holloschi A, Knoch TA, Auerswald EA, Welsh K, Reed JC (2002). Ionomycin-activated calpain triggers apoptosis. J. Biol. Chem.

[10] Squier MK, Miller AC, Malkinson AM, Cohen JJ (1994). Calpain activation in apoptosis. J. Cell Physiol.

[11] Leytin V, Allen DJ, Mykhaylov S, Lyubimov E, Freedman J (2006). Thrombin-triggered platelet apoptosis. J. Thromb. Haemost.

[12] Leytin V, Freedman J (2003). Platelet apoptosis in stored platelet concentrations and other models. Transfus. Apher. Sci.

[13] Vanags DM, Orrenius S, Aguilar-Santelises M (1997). Alterations in Bcl-2/Bax protein levels in platelets form part of an ionomycin-induced process that resembles apoptosis. Br. J. Haematol.

[14] Leytin V, Allen DJ, Mykhaylov S, Mis L, Lyubimov EV, Garvey B, Freedman J (2004). Pathologic high shear stress induces apoptosis events in human platelets. Biochem. Biophys. Res. Commun.

[15] Mason KD, Carpinelli MR, Fletcher JI, Collinge JE, Hilton AA, Ellis S, Kelly PN, Ekert PG, Metcalf D, Roberts AW, Huang DCS, Kile BT (2007). Programmed anuclear cell death delimits platelet life span. Cell.

[16] Leytin V, Allen DJ, Mutlu A, Mykhaylov S, Lyubimov E, Freedman J (2008). Platelet activation and apoptosis are different phenomena: Evidence from the sequential dynamics and the magnitude of responses during platelet storage. Br. J. Haematol.

[17] Li S, Wang Z, Liao Y, Zhang W, Shi Q, Yan R, Ruan C, Dai K (2010). The glycoprotein Ib alpha-von Willebrand factor interaction induces platelet apoptosis. J. Thromb. Haemost.

[18] Wang Z, Li S, Shi Q, Yan R, Liu G, Dai K (2010). Calmodulin antagonists induce platelet apoptosis. Thromb. Res.

[19] Wang Z, Shi Q, Li S, Du J, Liu J, Dai K (2010). Hyperthermia induces platelet apoptosis and glycoprotein Ib alpha ectodomain shedding. Platelets.

[20] Jobe SM, Wilson KM, Leo L, Raimondi A, Molkentin JD, Lentz SR, Paola JD (2008). Critical role for the mitochondrial permeability transition pore and cyclophilin D in platelet activation and thrombosis. Blood.

[21] Schoenwaelder SM, Yuan Y, Josefsson EC, White MJ, Yao Y, Mason KD, O’Reilly LA, Henley KJ, Ono A, Hsiao S (2009). Two distinct pathways regulate platelet phosphatidylserine exposure and procoagulant function. Blood.

[22] Shcherbina A, Remold-O’Donnell E (1999). Role of caspase in a subset of human platelet activation responses. Blood.

[23] Wolf BB, Green DR (1999). Suicidal tendencies: Apoptotic cell death by caspase family proteinases. J. Biol. Chem.

[24] Wolf BB, Goldstein JC, Stennicke HR, Beere H, Amarante-Mendes GP, Salvesen GS, Green DR (1999). Calpain functions in a caspase-independent manner to promote apoptosis-like events during platelet activation. Blood.

[25] Lin KH, Hsiao G, Shih CM, Chou DS, Sheu JR (2009). Mechanisms of resveratrol-induced platelet apoptosis. Cardiovasc. Res.

[26] Wang Q, Luo W, Zhang W, Dai Z, Chen Y, Chen J (2007). Iron supplementation protects against lead-induced apoptosis through MAPK pathway in weanling rat cortex. Neurotoxicology.

[27] Oda A, Druker BJ, Ariyoshi H, Smith M, Salzman EW (1993). pp60src is an endogenous substrate for calpain in human blood platelets. J. Biol. Chem.

[28] Nakagawa T, Yuan J (2000). Cross-talk between two cysteine protease families. J. Cell Biol.

