# Supplementary material for: Calpain Activator Dibucaine Induces Platelet Apoptosis
Source: Int J Mol Sci. 2011 Mar 25;12(4):2125–37. doi: 10.3390/ijms12042125 (PMC3127107; doi:10.3390/ijms12042125)

Supplementary Figure I. Dibucaine is a calpain-specific activator. Washed platelets were pre-treated with calpain inhibitor MDL28170 (100 μmol/L), calpain inhibitor I (100 μmol/L), calpain inhibitor II (250 μmol/L), and DMSO at room temperature (RT) for 10 min, respectively, and then were further treated with dibucaine (500 μmol/L) at RT for 15 min and subjected into western blot analysis using anti-filamin A antibody.

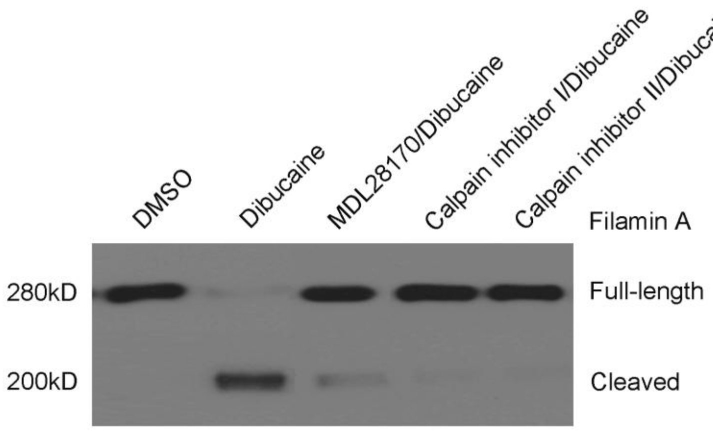

Supplementary Figure II. Effects of calcium chelators and caspase-3 inhibitors on dibucaine-induced cleavage of filamin A. Washed platelets were pre-treated with caspase-3 inhibitor z-DEVD-fmk (50 μmol/L), control DMSO, and EGTA (3 mmol/L) at RT for 15 min, respectively, and then were further treated with dibucaine (500 μmol/L) at RT for 15 min and subjected into western blot analysis using anti-filamin A antibody.

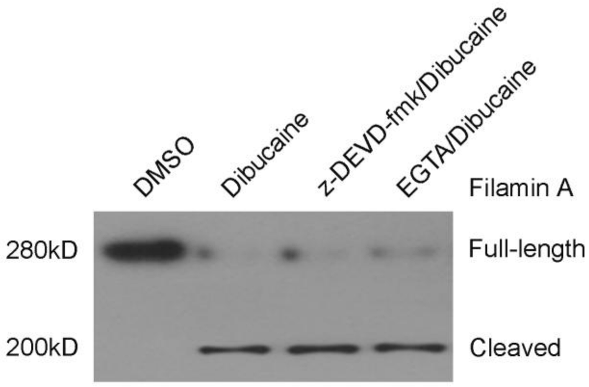

Supplementary Figure III. The caspase-3 inhibitor z-DEVD-fmk significantly inhibited PS exposure. Washed platelets were pre-treated with caspase-3 inhibitor z-DEVD-fmk (50 μmol/L) or control DMSO at RT for 15 min, and then were further incubated with dibucaine (500 μmol/L) at RT for 15 min and subjected into PS exposure analysis. Mean ± SD of the percentage of PS-positive platelets from three independent experiments is shown. * $P < 0.05$ compared with DMSO. ** $P < 0.01$ compared with DMSO. # $P < 0.01$ represents the comparison between z-DEVD-fmk/Dibucaine and Dibucaine.

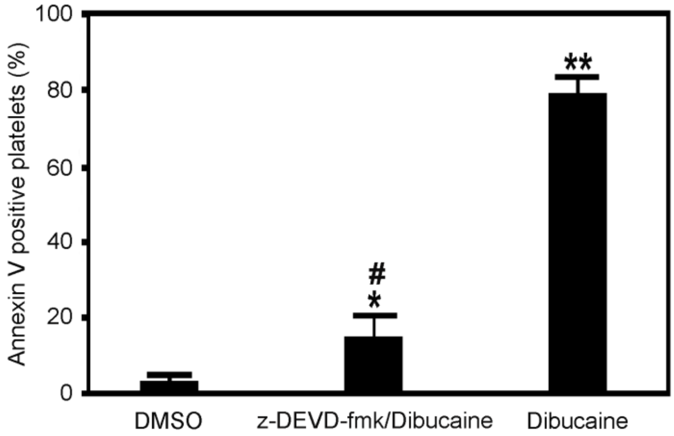

Supplement: Supplementary file 1 [file ijms-12-02125-s001.pdf]
